# The onset risk of carcinoma in patients continuing tacrolimus topical treatment for oral lichen planus: a case report

**DOI:** 10.1007/s10266-016-0255-4

**Published:** 2016-07-01

**Authors:** Mayu Morita, Seiji Asoda, Kazuyuki Tsunoda, Tomoya Soma, Taneaki Nakagawa, Masayori Shirakawa, Hirofumi Shoji, Hisao Yagishita, Takeji Nishikawa, Hiromasa Kawana

**Affiliations:** 1 Division of Oral and Maxillofacial Surgery, Department of Dentistry and Oral Surgery, School of Medicine, Keio University, 35 Shinanomachi, Shinjuku-ku, Tokyo, 160-8582 Japan; 2 Department of Oral Surgery, Shizuoka City Shimizu Hospital, Shizuoka, Japan; 3 Oral and Maxillofacial Surgery, Nippon Dental University Hospital, Tokyo, Japan; 4 Division of Oral Diagnosis, Dental and Maxillofacial Radiology and Oral Pathology Diagnostic Services, Nippon Dental University Hospital, Tokyo, Japan; 5 Samoncho Dermatological Clinic, Tokyo, Japan

**Keywords:** Oral lichen planus, Squamous cell carcinoma, Tacrolimus, Lip

## Abstract

Oral lichen planus is a chronic inflammatory mucocutaneous disease. Topical use of steroids and other immuno-modulating therapies have been tried for this intractable condition. Nowadays, tacrolimus ointment is used more commonly as a choice for treatment. However, a number of discussions have taken place after tacrolimus was reported to be carcinogenic. This report describes a patient who applied tacrolimus ointment to the lower lip after being diagnosed with oral lichen planus in 2008, and whose lesion developed squamous cell carcinoma in 2010. Since the relationship between tacrolimus and cancer development has been reported in only a few cases, including this case report, the clinician must be careful selecting tacrolimus as a second-line treatment for oral lichen planus.

## Introduction

Oral lichen planus (OLP) is a chronic mucocutaneous disease, and the etiology of OLP has been reported that stress, systemic medications, dental materials, chronic liver disease, hepatitis C virus and graft-versus-host disease have been put forward to explain the pathogenesis [1]. It has been reported that this intractable disease has a 0.1–4 % risk of malignant transformation [2]. Up until now, the topical use of steroids and other immuno-modulating therapies have been the standard treatment for OLP.

Since 1999, when Vente et al. [3] reported the validity of tacrolimus ointment for OLP, a number of pilot studies have been performed [4–6]. Tacrolimus, also called FK506, is a macrolide immunosuppressant produced by *Streptomyces tsukubaensis* and has similar effects as cyclosporin A. It acts by inhibiting calcineurin, a ubiquitous calcium-dependent protein phosphatase that is responsible for immune response [7]. However, in recent years there have been a number of discussions held after reports regarding its carcinogenicity appeared.

We hereby report a case of a patient with OLP who, after using tacrolimus ointment for 14 months, developed squamous carcinoma of the lower lip.

## Case report

**Fig. 1:**
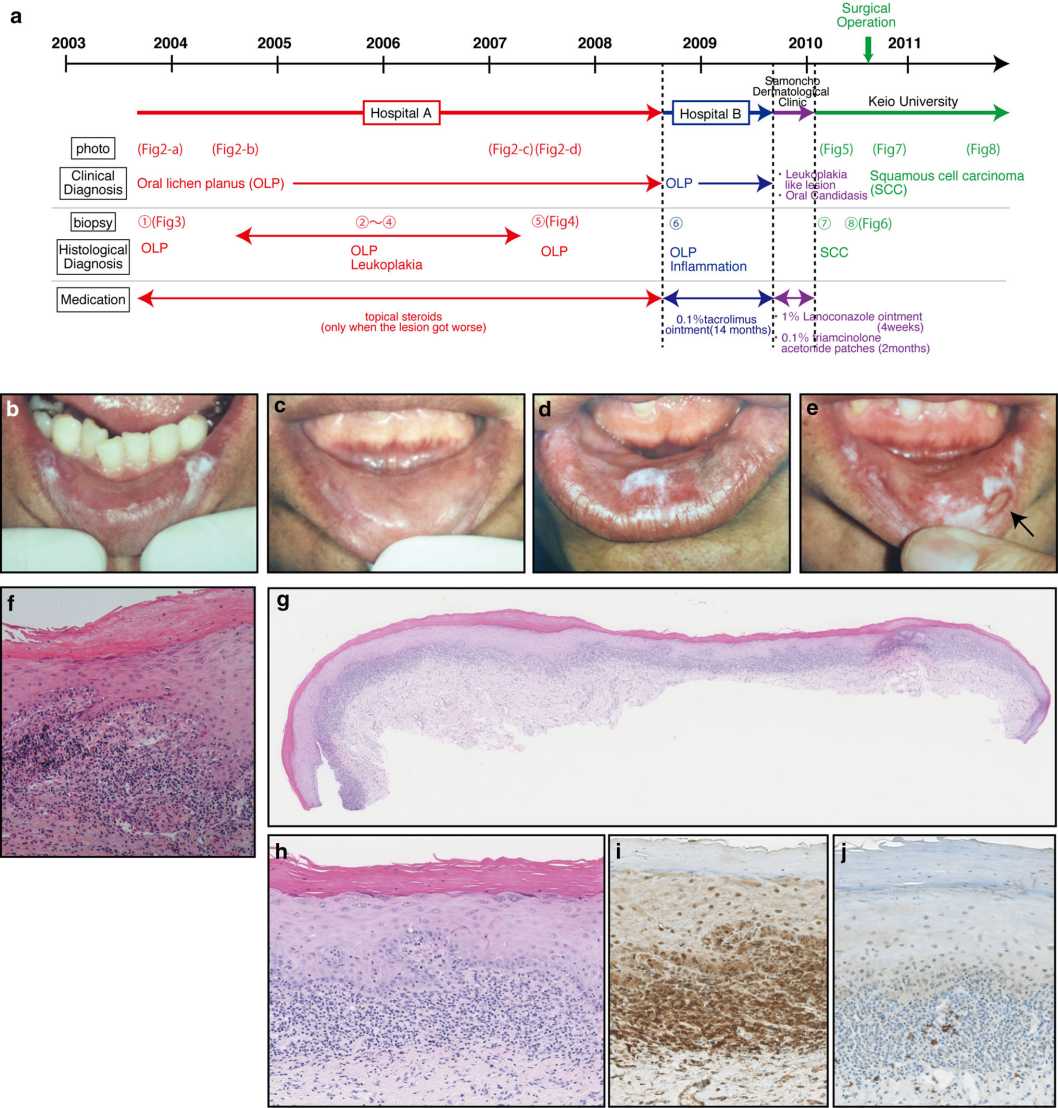
**a** Summarized schema of the course. Clinical appearances of the lesion from 2003 to 2007. **b** Photograph taken in October 2003. *White* lesions seen on both sides of the lower lip. **c** Photograph taken in August 2004. The surface appearance of the lower lip returned to normal. **d** Photograph taken in March 2007. The *white* spot of the lesion appeared again on the *left* *side* of the lower lip. **e** Photograph taken in June 2007. Biopsy was performed in 2007 from *left* *side* of lower lip (indicated by an *arrow*). **f** A biopsy in 2003 from the lower lip suggests “oral lichen planus with mild dysplasia from reactive” biopsy specimen showing band-like infiltration of lymphocytes at the dermo-epidermal interface (HE stain, original magnification ×100). A biopsy in 2007 from the lower lip suggests “oral lichen planus.” **g** Loupe imaging stained with HE. Enhancement of partial cornified layer of the mucosal epithelial layer and typical band-like lymphocytic infiltrate were seen in this section. **h** High magnification of **g** (HE stain, high magnification ×20). Infiltration of lymphocytes in the epithelial layer, and mild liquefactive degeneration were seen in this section. **i** Immunostaining using anti-CD3 antibody. **j** Immunostaining using anti-CD20 antibody

In October 2003, a 63-year-old male patient was referred to the Department of Oral and Maxillofacial Surgery at a regional hospital (Fig. 1a, Hospital A) for evaluation of a white slightly eroded lesion with white streaks on the lower lip (Fig. 1b). The patient had a history of hypertension and an adrenal gland tumor and was an occasional social drinker, but had no history of smoking and reported no allergies. The first biopsy was taken in October 2003, and the histopathological diagnosis was “oral lichen planus” (Fig. 1f). The patient had dental silver fillings in molar teeth, but did not have any intraoral lesions consistent with OLP except on the lower lip. Topical steroids were first used to treat OLP at the first hospital (Fig. 1a, Hospital A). Because the clinical appearance of the lesion continued to change among visits (Fig. 1c–e), biopsies were taken 4 times from March 2004 to March 2007 at Hospital A. The histopathological diagnosis was either “oral lichen planus” or “hyperkeratosis without dysplasia,” but the clinical appearance of the lesion became gradually worse. The reason why we diagnosed it as OLP in histological section is: (1) the typical band-like lymphocytic infiltrates were seen in the whole section (Fig. 1g). (2) Epithelial surface hyperkeratosis or hyperparakeratosis was seen. (3) Civatte bodies were seen in the bottom of epithelial or lamina propria (Fig. 1h). (4) The histological patterns in B cell and T cell. B cells and plasma cells are infrequent in OLP, and in this case, we could not observe B cells, because plasma cells were not observed in HE staining (Fig. 1h). Immunostaining was performed, and the results showed most lymphocytes were positive to CD3; however, only few CD20 positive cells were observed (Fig. 1i, j). The patient used topical steroids at Hospital A (Fig. 1a) when the lesion got worse. We did not diagnose as oral lichenoid lesions (OLL), because B cell follicles, also known as lymphoid follicles, were not observed in histological section.

In August 2008, the patient was referred to another hospital (Fig. 1a, Hospital B), Department of Dentistry, School of Medicine in Tokyo, since the clinical appearance was clearly becoming worse. The clinical diagnosis made there was again OLP, and the patient was given tacrolimus 0.1 % ointment for topical application. The patient used the topical ointment intermittently based on the condition of his lesion for 14 months. Tacrolimus was not used per the recommendations that are shown in Table 1, because the patient tried 3 times a day to speed up the cure. The surface appearance of the lower lip returned to normal when he used tacrolimus, but the stinging sensation remained. Again the symptoms got worse when the patient stopped using the ointment, so the patient had to continue using it for 14 months. A biopsy was taken from the lower lip when the lesion got worse again when the tacrolimus treatment was terminated in 2009, and the histopathological report was not diagnostic and described as “inflammation, and no malignancy is seen.”

**Table 1:** Information for healthcare professionals: tacrolimus ointment (FDA) [13]

Apply a thin layer of tacrolimus ointment twice daily to the areas of skin affected by eczema
Avoid getting tacrolimus ointment in the eyes or mouth. Do not swallow tacrolimus ointment
Do not use ultraviolet light therapy, sun lamps, or tanning beds during treatment with tacrolimus ointment
Use tacrolimus ointment only as a second-line agent for short-term and intermittent treatment of atopic dermatitis, a form of eczema, in patients unresponsive to, or intolerant of other treatments
Use tacrolimus ointment only for short periods of time, not continuously. The long-term safety of tacrolimus ointment is unknown
Use the minimum amount of tacrolimus ointment needed to control the patient’s symptoms. In animals, increasing the dose resulted in higher rates of cancer

**Fig. 2:**
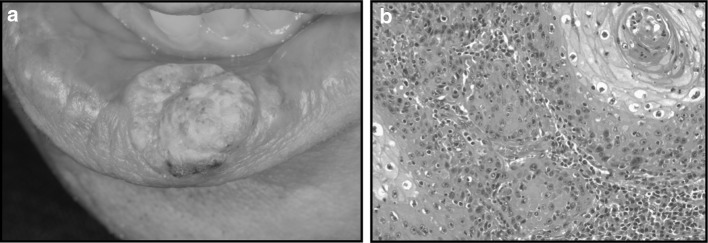
**a** Photograph taken in January 2010. The ulcerated lesion of the lower lip (22 × 18 × 8 mm) with indurated borders. **b** A biopsy from the lower lip suggests “well-differentiated squamous cell carcinoma.” A horn pearl is prominent in the *right* *part of the image* (HE stain, original magnification **a** ×40; **b** ×100)

In September 2009, the patient visited a dermatology specialist at Samoncho Dermatological Clinic, Tokyo, because the lesion did not improve, even though the tacrolimus ointment had been applied to the lower lip. The stinging sensation was still present. When the patient visited to Samoncho Dermatological Clinic for the first time, the tacrolimus treatment was terminated. Wet scrapings from the lower lip mounted in 15 % potassium hydroxide (KOH preparation) revealed pseudomycelia and blastospores, suggesting oral candidiasis. The patient was given 1 % lanoconazole ointment for 4 weeks, which healed the lesion on the lower lip. Then, 3 small infiltrated lesions with rough surfaces appeared on the lower lip, where 0.1 % triamcinolone acetonide patches were applied for 2 months. At the following visit in January 2010, the left lower lip had worsened to grow into an ulcerated papillomatous nodule on its left side. Because of these changes in the lesion, the patient was finally referred to the Department of Oral and Maxillofacial Surgery, Keio University School of Medicine, in January 2010. The ulcerated lesion was 22 × 18 × 8 mm with indurated borders, and white spots were found around the periphery (Fig. 2a). Computed tomography and magnetic resonance imaging were performed, but it only showed exophytic lesions on the lower lip. A biopsy was taken from the exophytic lesion, and the histopathological diagnosis was “well-differentiated squamous cell carcinoma” (Fig. 2b). Positron emission tomography–computed tomography was then performed, and the tumor was classified as T2N0M0. The patient was given the choice of radiation therapy or surgery, and the patient chose to undergo surgery. The extent of resection of the lesion from the tumor was about 10 mm, and a white lesion around 5 mm was removed. Both flaps were designed as V-Yflap, and red lips used a pedicle flap of buccal mucosa on both sides. Five years have passed since the operation, and there has been no recurrence. The patient is satisfied and has no complaints regarding the aesthetics and the functional capabilities of the lower lip.

## Discussion

OLP is a chronic inflammatory mucocutaneous disease, and topical use of steroids and other immuno-modulating therapies have been used for this intractable condition. Nowadays, tacrolimus ointment is used more commonly and seems effective as a second choice to treat it [8]. The question in this case lies in whether OLP was diagnosed properly in the early stages. As Jing-Ling Xue et al. reported, most patients had multiple oral sites of involvement; the disease was confined to the lower lips in only 60 patients out of 674 patients with OLP [9]. In the case of this patient, the lesion on the lower lip could not be firmly diagnosed as OLP in clinical appearance, which was the reason why a biopsy was performed several times.

Currently, OLL is considered by some to put the lesion at risk of malignant transformation [10]. In a recent study, it was shown that all cases of malignant transformation showed cases of OLL but not OLP; however, both lesions are likely to have a risk of malignant transformation. Very recently, Fitzpatrick et al. reported about oral lichenoid mucositis and cancer development and discussed the difficulties in evaluating lichenoid responses in oral lesions with or without dysplasia [11]. The potential for malignant transformation is still controversial. We also think that while OLP, which was the diagnosis from 2003 to 2007, did not become malignant directly in this case, OLL may follow after the use of tacrolimus ointment.

Tacrolimus ointment is known to cause both skin cancers and lymphoma in humans by suppressing the body’s normal immune defenses against cancer. In animals, increasing the dose resulted in higher rates of cancer. Tacrolimus ointment is sometimes absorbed through the skin, although usually at very low amounts. In case of OLP, the blood concentration absorbed from the oral mucosa is reported between lower than 1.3–9.6 ng/ml [12]. This number is lower than 20 ng/ml, the safe blood concentration for the body using tacrolimus after a kidney transplantation [13]. A pharmaceutical company producing tacrolimus in Japan has received 21 post-marketing reports linking tacrolimus ointment with cancer-related adverse effects, including 16 lymphomas, 3 cutaneous tumors, and 2 other tumor types. On the other hand, it is reported that the risk of cancer while using tacrolimus is lower than the risk of cancer naturally developing (i.e., not using immunosuppressants) [14].

The Food and Drug Administration (FDA) issued a public health advisory in June 2006. The advice to physicians is shown in Table 1. The British Society of Oral Medicine issued guidelines for the management of OLP in 2010 [15].

In this case as in others, OLP might have transformed to SCC naturally, as already reported [10]. However, the tumor developed from the lower lip where the patient applied the tacrolimus ointment, and as such we could not rule out the relationship between the long-term use of tacrolimus and cancer development. In addition, until distinct clinical and histological criteria have been developed on how to differentiate OLP from OLL, both lesions have to be considered as at risk of malignant transformation.
